# Integrated Care to Address the Physical Health Needs of People with Severe Mental Illness: A Mapping Review of the Recent Evidence on Barriers, Facilitators and Evaluations

**DOI:** 10.5334/ijic.2605

**Published:** 2018-01-25

**Authors:** Mark Rodgers, Jane Dalton, Melissa Harden, Andrew Street, Gillian Parker, Alison Eastwood

**Affiliations:** 1 Centre for Reviews and Dissemination, University of York, Heslington, YO10 5DD, York, UK; 2 Department of Health Policy, London School of Economics and Political Science, WC2A 2AE, London, GB; 3 Social Policy Research Unit, University of York, Heslington, YO10 5DD, York, UK

**Keywords:** integrated care, mental health, physical health, mapping review

## Abstract

People with mental health conditions have a lower life expectancy and poorer physical health outcomes than the general population. Evidence suggests this is due to a combination of clinical risk factors, socioeconomic factors, and health system factors, notably a lack of integration when care is required across service settings.

Several recent reports have looked at ways to better integrate physical and mental health care for people with severe mental illness (SMI). We built on these by conducting a mapping review that looked for the most recent evidence and service models in this area. This involved searching the published literature and speaking to people involved in providing or using current services.

Few of the identified service models were described adequately and fewer still were evaluated, raising questions about the replicability and generalisability of much of the existing evidence. However, some common themes did emerge. Efforts to improve the physical health care of people with SMI should empower staff and service users and help remove everyday barriers to delivering and accessing integrated care. In particular, there is a need for improved communication among professionals and better information technology to support them, greater clarity about who is responsible and accountable for physical health care, and greater awareness of the effects of stigmatisation on the wider culture and environment in which services are delivered.

## Introduction

People with severe mental illness (SMI; such as schizophrenia, schizotypal and delusional disorders; bipolar affective disorder; severe depressive episode(s) with or without psychotic episodes) [1] have a lower life expectancy and poorer physical health outcomes than the general population [2]. Evidence suggests this discrepancy is driven by a combination of clinical risk factors (e.g., comorbid diabetes, cardiovascular disease), socioeconomic factors, and health system factors, notably a lack of integration when care is required across service settings [3]. There is fragmentation in how care is co-ordinated between family doctors and hospitals, between physical and mental health care, and across health and social care.

Physical health and mental health are closely linked, and demands have been repeatedly placed on health services to deliver an equal response to the treatment of each [4,5]. Even so, many patients with SMI remain under-served. While our work was intended to inform the English National Health Service, and our discussion of current policy draws on English examples, the issues identified are ones that are being grappled with internationally [6].

Services for mental health conditions have traditionally been separate from those for physical conditions but there is increasing emphasis internationally on developing a whole system approach to improve integration between the two, with particular focus on patient-centred development and delivery [4,7]. This is not new; a focus on patient-centred delivery of health services for people with severe mental illness has been advocated for many years. In the UK, ‘The Care Programme Approach (CPA)’, originally introduced in 1991 and updated in 2013 [8] is a national system setting out how secondary mental health services should help people with severe mental illness and related complex needs. Those eligible for CPA are entitled to a full assessment of health and social care needs, a care plan (overseen by a care co-ordinator) and regular reviews of health and progress, although health care providers are not required to follow this guidance and could adopt their own policy. The personalisation agenda for people with SMI also featured in the National Service Framework for Mental Health in 1999 [9]. Similar approaches to care-coordination have been implemented internationally [10,11,12,13,14,15,16].

In 2006, the English Department of Health produced a commissioning framework for supporting the physical needs of people with SMI [2]. This described the nature of pilot health improvement programmes in which a lead mental health nurse practitioner attached to an existing team (e.g., primary care team or community mental health team) would be responsible for conducting physical health checks, in-depth consultations (including providing relevant information and exploring broader health-related issues such as employment or education), referral to screening and health promotion services, and establishing specific one-to-one or group health improvement interventions. The pre-requisites for this type of programme were defined [2] and evaluations have emerged since [17,18]. However, there is little evidence of their wider implementation.

Existing guidance and incentives to address the treatment and management of people with SMI include NICE guidelines for various mental health disorders,[19,20,21,22] and an incentive for secondary health care providers to improve the physical health care of people with SMI (a Commissioning for Quality and Innovation payment framework - CQUIN) [23]. This CQUIN helps ensure service users have their physical and mental health diagnoses recorded, and aims to promote effective communication between primary care, specialist mental health services and service users. In addition, a recent proposal by NICE to improve the quality of care by family doctors considers the introduction of new quality indicators to identify and support people with SMI who are at risk of cardiovascular disease [24].

In 2013, the Mental Health Foundation (MHF) undertook an inquiry into integrated care. This substantial piece of work was based on a literature review on integrated health care and mental health care, three expert seminars attended by 31 people and a call for evidence on the best ways to integrate care which led to over 1200 responses. The scope of the enquiry incorporated both health and social care and identified a number of structural and organizational arrangements at the heart of good integrated care for people with mental health problems, including information sharing systems, shared protocols, joint funding and commissioning, and co-location of services [25]. In the same year, a systematic review found just four evaluations of interventions that integrated medical and mental health care to improve medical outcomes in individuals with SMI (Bradford et al) [26]. This review found evidence that some interventions were associated with increased immunisation and screening rates, with mixed results on physical functioning, and an absence of preventative or chronic medical care measurement.

It is against this background that we reviewed the most recent evidence and service models designed to better integrate physical and mental health care for people with SMI. The latest initiatives in practice aim to improve integrated care for people with SMI when they access services for an acute or chronic physical health condition (eg, assistance from a liaison psychiatrist in the Emergency Department) [27]. But there is less information about integrated care initiatives addressing the physical health needs of people with SMI when they enter mental health services (e.g., how their physical health is attended to in a psychiatric hospital or specialist mental health unit), so this was our focus.

## Theory and methods

Our aims were to explore what current provision exists, and to map the most recent evidence about if and how service models of care address the physical health needs of people with mental health problems, primarily within mental health service settings. The research was designed as a rapid review to identify, appraise and synthesise relevant evidence from 2013 to 2015, including an update of the comprehensive review by Bradford et al (2013), bringing this together with grey literature and insights from an expert advisory group.

The focus of the review was health care services that included steps to address the physical health needs of people diagnosed with SMI when delivered in a mental health care setting. The review was funded by the UK National Institute for Health Research (NIHR), with a full-length report focusing on the implications for UK health services was produced for the NIHR Health Services and Delivery Research programme [28]. This journal article provides an accessible adaptation of the NIHR review for an international audience by mapping and summarising the identified evidence.

We defined SMIs to include: schizophrenia, schizotypal and delusional disorders; bipolar affective disorder; and severe depressive episode(s) with or without psychotic episodes [1]. We defined physical health outcomes broadly, including the assessment and modification of cardio-metabolic risk factors, anthropometric measures, and physical functioning.

We established an advisory group of field experts and of service users identified through local contacts who provided helpful signposting to recent literature and service models. We also engaged with advisers in detailed face-to-face or teleconference conversations. While the literature review was international in focus, all Advisory Group members were patients or professionals with relevant experience in the National Health Service in England.

The literature search was undertaken to identify empirical and descriptive publications relating to integrated care for the physical health of people with SMI. We carried out searches to find and prioritise any new evaluative studies since 2013, using an adapted version of the search strategy from the 2013 Bradford et al review. Nine electronic databases were searched from 1st January 2013 to May/June 2015. Further searches were undertaken to identify UK and international guidelines and any relevant English-language government policy documents from the UK, Australia, New Zealand, Canada or USA. The project team also collected relevant literature recommended by members of the Advisory Group.

## Evidence selection criteria and procedures

Evidence identified from the literature searches were selected based on the relevance of their population, intervention, outcome, study design, and setting (see below for critieria). Three reviewers independently selected studies for inclusion. One reviewer extracted relevant data, the accuracy of which was checked by a second reviewer. Disagreements were resolved by discussion or with the involvement of a third party.

Population: People diagnosed with SMI.

Intervention: Any health care services that include arrangements to address the physical health needs of people with SMI. Programmes primarily concerned with the organisation and delivery of services rather than the implementation of discrete health technologies.

Outcome: Any outcome relevant to the provision and implementation of integrated care. For the evaluative literature, outcomes were restricted to those related to physical health (including sexual health).

Study design: Empirical and descriptive publications, including evaluative studies; policy/guideline documents.

Setting: Integration of services primarily within the healthcare sector. Models focused on the wider integration of services spanning non-health care settings (e.g., social care, education, employment, housing, and voluntary sector provision) were not eligible for inclusion.

We carried out a narrative synthesis, building on the 2013 MHF inquiry [25]. The inquiry incorporated both health and social care, and identified nine structural and organizational arrangements at the heart of good integrated care for people with mental health problems. We used these nine facilitators as a guiding framework to explore the elements of interventions or care models. These were:

1. Information sharing systems
2. Shared protocols
3. Joint funding and commissioning
4. Co-location of services (e.g., services brought together for physical and practical ease of access)
5. Multidisciplinary teams
6. Liaison services (e.g., provision of shared expertise across service settings)
7. Navigators (e.g., named care co-ordinators)
8. Reduction of stigma
9. Research (e.g., to ascertain the best way of delivering and evaluating integrated care)

This article provides an overview of the recent literature, including references to the original studies to allow the reader to undertake further in-depth reading on interventions of specific interest.

## Results

We identified 45 publications describing 36 separate approaches to integrating physical health needs into the care of people with SMI for inclusion (see Table 1). These comprised a range of study designs including systematic and non-systematic literature reviews, primary studies, book chapters, conference abstracts, dissertations, policy and guidance documents, feasibility studies, descriptive reports and programme specifications. Twenty-seven papers reported on 25 distinct evaluations of programmes or interventions.

**Table 1:** Classification of included publications.

	Evidence type	1. Information sharing systems	2. Shared protocols	3. Joint funding and commissioning	4. Co-location of services	5. Multidisciplinary teams	6. Liaison services	7. Navigators	8. Research	9. Reduction of stigma

Bartels [10]	E				•			•		
Bellamy [29]	E			•	•	•	•	•		
Bradford [26]	E	•	•	•	•	•	•			
Chawstiak [30,31]	E				•	•	•			
Curtis [32]	E				•	•				
De Hert [33]	P		•							
Department of Health [2]	P, E	•			•	•	•			
Department of Health [4]	P			•		•				
Druss [11]	E				•	•	•			
Greater Manchester CLAHRC [34,35]	E	•	•			•	•	•		
Happell [12,36,37,38]	D, E				•		•	•		•
Hardy [39]	E									
Jones [40]	E							•		
Kelly [13] Brekke [41]	E							•		•
Kern [14]	D	•	•	•	•	•	•	•	•	•
Kilany [42]	E	•	•	•	•	•	•	•	•	•
Kilbourne [43,44,45,46]	D, E	•					•			•
Lee [47]	E				•	•				
Maki [48]	E	•				•			•	
Mental Health Foundation [25]	D	•	•	•	•	•	•	•	•	•
NHS IQ [49]	E			•		•		•		•
NHS London [50]	D		•							
Nover [15]	D				•	•		•		
Parks [16]	D	•	•	•	•	•	•	•	•	•
Pirraglia [51]	E				•					
Rubin [52]	E				•	•	•	•		
Shackleford [53]	D				•		•			
Solomon [54]	D							•		
Stark [55]	D	•					•		•	
Tallian [56]	D		•							
Ungar [57]	D				•	•	•			
Vanderlip [58]	D					•		•		
Vinas Cabrera [59]	E	•	•							
Von Esenwein [60]	E	•								
Welthagen [61]	D				•		•			
Yeomans [62]	E	•								

• 1–9 indicates components of the intervention, according to the nine factors of good integrated care. E = Evaluation; P = Policy document; D = Descriptive (non-evaluative) publication.

Most service models were multi-component programmes incorporating two or more of the factors previously identified as facilitators of integrated care [25]. However, with the exception of ‘navigator’ approaches, underlying models or theories were rarely articulated in the evidence. The majority of programmes were in community and/or secondary care mental health settings in the UK, North America, or Australia. Few were described in great detail and fewer still were comprehensively evaluated, raising questions about the replicability and generalisability of much of the existing evidence. However, the studies provided insight into the presence or use of the nine facilitators identified by the MHF, and we have used these to frame our results.

### Information sharing systems [2,14,16,25,26,34,35,42,43,44,45,46,48,55,59,60,62]

The MHF inquiry identified the need for a compatible information system within and across different care organisations that could establish individual electronic records of service users’ integrated health and social care needs and interventions. Being able to access information from single or multiple electronic medical records (EMRs) is an important facilitator, as it allows providers to identify and track SMI populations and individuals needing physical health services [16].

Both the UK general medical services (GMS) contract, the US Substance Abuse and Mental Health Services Administration’s (SAMHSA) Primary and Behavioural Health Care Integration (PBHCI) funding programmes incentivize a registry to track the primary care needs of, and outcomes for, people with serious mental illness [14,63,64]. The collection and maintenance of such information necessarily requires an adequate IT infrastructure. However, PBHCI grantees have noted both technical and legal barriers to implementing the required shared information systems. For example, Web-based registry software has thus far proved to be inadequate, resulting in organisations relying on less useful paper or Excel-based versions [14].

Being able to access information from single or multiple electronic medical records (EMRs) is an important facilitator, as it allows providers to identify and track patient SMI populations and individuals needing physical health services [16]. However, behavioural health care providers in some US States have been prevented from being able to share EMRs as a consequence of federal privacy laws regarding drug and alcohol information. Regulatory barriers that limit information exchange between primary and mental health care have been identified as particularly problematic [30]. It is not clear from the published evidence to what extent such barriers have been overcome by self-contained US funding systems, such as the Veterans’ Health Administration (VA), where integrated registry and EMR data have been used to target the physical health care needs of people with SMI [11,26,43,44,45,46].

Some authors have proposed allowing patients to opt-in to release health information into the shared system to overcome medico-legal barriers,[60] though this may raise questions about informed consent, particularly among SMI populations. In the UK, the Data Protection Act (1998) and the Human Rights Act (1998) govern the sharing and confidentiality of health records and the Health and Social Care Information Centre (2013) has produced guidance on handling confidential information [65].

### Shared protocols [14,16,25,26,33,34,35,42,50,56,59]

Shared protocols between two or more organisations, or parts of an organisation, set out the responsibilities of each in delivering an agreed service and/or outcome. The MHF inquiry was broadly supportive of shared protocols within and between the organisations that support people with mental health problems [25]. A major theme to emerge from the more recent literature and from our advisory group was the importance of responsibility and accountability [34,35]. Two field experts felt that there is currently insufficient clarity about who is responsible for the physical health needs of people with SMI. Both mentioned the physical health care of SMI patients falling to secondary care for first 12 months post-diagnosis, followed by (where clinically appropriate) transfer of responsibility to primary care, in line with the shared care arrangements outlined in published quality standards [66]. However, several advisory group members also mentioned an ongoing lack of clarity and/or disagreement about roles and responsibilities (“Everyone thinks it is someone else’s business”). The wider literature suggests that maintaining absolute clarity about who is responsible for each aspect of physical health care is difficult but crucial to the success of integrating physical and mental health care.

Specific protocols have been developed for the assessment and management of the cardiometabolic health in people experiencing psychosis and schizophrenia, [67] [33] though these differ in their recommendations about which care provider should be responsible for longer term monitoring and care coordination.

### Joint funding and commissioning [4,14,16,25,26,29,42,49]

The MHF inquiry concluded that separate funding streams hinder integrated care, while pooled funding and services commissioned across boundaries increase the likelihood of service users receiving better care [25]. A recent review of 38 schemes that integrated health and social care funds suggested that improved integrated care tends to uncover unmet needs, with total care costs likely to rise. Nevertheless, better integration may still offer value for money if additional costs are offset by improvements in quality of life [68].

Much of the US literature has focused on overcoming funding barriers in the provision of collaborative stepped care. This has recently included the provision of integrated primary care services for people with SMI within Community Mental Health Centre (CMHC) settings, funded through the Substance Abuse and Mental Health Services Administration’s (SAMHSA) Primary and Behavioural Health Care Integration (PBHCI) programme. However, alternative administrative arrangements can include global payment systems for physical, mental, and dental care for Medicaid beneficiaries (via coordinated care organisations; CCOs) and self-contained systems (Veterans Health Administration, Department of Defense, private insurers) [11,14,26]. While the organization of services may vary across PBHCI grantees, receipt of funding is contingent on CMHCs establishing a formal link with a primary care partner.

Some of the problems noted in the US literature – such as insurance companies refusing to pay for lipid panel orders for patients not taking second-generation anti-psychotics [48] – may not be directly relevant to the UK, but such observations highlight how fragmented funding can undermine the implementation of integrated care programmes.

### Co-location of services [2,10,11,12,14,15,16,25,26,29,30,31,32,36,37,38,42,47,51,52,53,57,61]

Co-location of primary care and specialist mental health staff could provide significantly improved integration of care for people with mental health problems; but only if the staff understood their roles and responsibilities and worked willingly and collaboratively together [25]. This emphasises that people rather than organisational systems or structures are primarily responsible for the successful integration of care. Much of the published evidence on co-located care identified through this mapping review was concerned with the primary care professionals providing clinics in community or inpatient mental health settings [10,11,14,30,51,53,57,61]. The literature also highlighted the need for willing, interested, committed and passionate staff [57] plus commitment from leaders and administrators [30]. The need to plan for and provide sufficient physical space for any primary care services to be located in a mental health clinic [14,15]. and for co-located care sites to be both highly visible and easily accessible (including open access arrangements that allow walk-in care for people with SMI) were also stressed [14,51].

### Multidisciplinary teams [2,4,11,14,15,16,25,26,29,30,31,32,34,35,42,47,48,49,52,57,58]

As acknowledged by the MHF report, the principles of multidisciplinary care are already well established in mental health services. However, although effective communication between multi-agency health professionals has long been acknowledged as necessary to improve the physical health of people with SMI, [2] both field experts and service users told us that communication often remains poor, particularly between primary and secondary care.

One UK pilot study attempted to overcome communication barriers through the work of a Community Physical Health Coordinator (CPHC), who would hold regular multi-disciplinary team meetings with GP practices (involving at least a GP, Practice Manager/Administrator, Practice Nurse/Health Care Assistant) to establish shared care with the local Community Mental Health Team (CMHT). In addition, the CPHC would hold a definitive list of lifestyle services and liaise with Practice Managers and GPs in between MDT meetings [34,35].

### Liaison services [2,11,12,14,16,25,26,29,30,31,34,35,36,37,38,42,43,44,45,46,52,53,55,57,61]

The MHF inquiry was strongly supportive of the concept of liaison services – both psychiatric liaison services in physical health care settings and physical health care in mental health settings [25]. Advisory group service users told us that they would like to know that there is someone with responsibility for the physical health needs of SMI service users, particularly in the inpatient setting. We also found several published descriptions of primary care clinics or placement of physical health practitioners in inpatient [52,61] and outpatient [11,14,36,37,38,53,57] mental health settings. Field experts also described existing services such as dedicated GP sessions on forensic wards and in-reach specialist diabetes nurses.

### Navigators [10,12,13,14,15,16,25,29,34,35,36,37,38,40,41,42,49,52,54,58]

The MHF inquiry supported the principle of a single named individual to help people navigate their way through complex health and social care systems [25]. However, while the advisory group suggested that continuity of care is particularly important for the SMI population, they said it is becoming increasingly rare within primary care. In response to this, further care models have incorporated a ‘navigator’ role. While navigators or care coordinators are generally implemented to negotiate the boundaries between health, social care, education, and housing sectors, this role can be just as important for helping people with SMI negotiate boundaries *within* heath care, between physical and mental health services, or between primary and secondary care [13,29,34,35,41,54].

Service users raised questions about the extent to which navigators should engage in advocacy for patients, particularly when dealing with services less accustomed to SMI (e.g., when people with SMI have dental care withdrawn due to missed appointments). One study reported specialist ‘Care Coordinators’ having insufficient authority to exert control over other care professionals to ensure care is properly integrated [69].

### Reduction of stigma [12,13,14,16,25,36,37,38,41,42,43,44,45,46,49]

Both service users and field experts from our advisory group reported that GPs and non-mental health specialists can appear reluctant to tackle severe mental illness. Some attributed this to the perception that the SMI population can be “troublesome” or excessively difficult to deal with, generally because of non-attendance of appointments and non-compliance with treatment advice. The MHF inquiry focused on the importance of education and training in mental health issues for the public and healthcare workforce [25]. The recent published literature has noted that primary care practitioners may be uncomfortable about and find it difficult to deal with the complexity and/or the slow pace of working with people SMI, relative to the wider primary care population [14]. A major concern raised both in the literature and among the advisory group was ‘diagnostic overshadowing’, whereby signs and symptoms of physical illness can be misattributed to severe mental illness, leading to under-diagnosis and mistreatment of the physical condition.

### Research [14,16,25,42,48,55]

The MHF inquiry called for more research into how best to support people with complex, co-morbid needs that addresses both the effectiveness and economic assessment of integrated care models [25]. However, most of the programmes identified through our update searches and contact with field experts have either not been evaluated, or only evaluated on a small-scale within a local context.

## Discussion

While our mapping review was focused on integrated care initiatives addressing the physical health needs of people with SMI when they enter mental health services, the themes emerging from the literature and advisory group consultations touched on concerns about continuity of care more broadly [70].

There is broad agreement about what needs to be done to improve the physical health of people with SMI, but not about who should be responsible, particularly within multidisciplinary teams involved in the co-ordination and provision of mental health services. Simply having an appropriate skill mix within a team is not a sufficient guarantee of integrated care. Within multidisciplinary teams, there must be clarity about the specific aspects of care for which individuals in the team are responsible and accountable, supported by effective communication between team members. Poor communication within teams and between providers might give rise to missed opportunities to intervene when needed.

Shared protocols, joint action plans and decision support tools may assist by clarifying responsibilities and supporting record keeping and communication across boundaries. Organisational incentives alone are likely to be inadequate unless professionals have the appropriate knowledge, skills, resources, and environment to work in. Instead, in order to assign responsibility, various ‘navigator’ models have been developed, in which a single professional takes primary responsibility for co-ordinating care across multiple settings. However, the handful of evaluations of these models tended to be superficial, with little clarity about implementation. That said, the available evidence suggests that any individual tasked with co-ordinating care needs to be empowered with the authority to influence other care professionals.

A fundamental requirement for successful integration of physical and mental health care is having the people with the appropriate skills and attitudes to deliver those services. Therefore, any planned structural changes should consider the likely impact on the attitudes, skills and behaviours of the people interacting within and across health organisations, be they health professionals or service users. Many factors identified as facilitators either empowered individuals and/or minimised the effort needed for individuals to provide and access integrated services.

Mental health professionals who avoid physical health actions through a lack of confidence in their own skills may be empowered through targeted training and greater clarity about their responsibilities in relation to physical health. Care co-ordinators/navigators may have an empowerment role by providing advocacy for service users with SMI in certain settings, and might benefit from greater formal authority over care integration. All health professionals need time for training and to collaborate on patient care, which can be difficult in clinical settings with heavy caseloads. Management commitment to protect time and resources for such activities has been raised as a worthwhile investment.

Integrated information systems and individual electronic records are seen as key to good integrated care but have yet to be properly implemented due to various technical, legal, and organizational barriers. However, these remain the most promising means of simplifying communication and collaboration among professionals across multiple services.

The literature also mentions simple measures such as informal referral procedures, high visibility of service locations, and open access arrangements as facilitators of physical health clinics for people with SMI in mental health settings. However, evaluation or wider dissemination of these local innovations was uncommon.

The Advisory Group described several ways in which the existing organization of services, and often unconscious assumptions, attitudes, and behaviours of health care staff, can be stigmatizing to people with SMI, leading to inattention to their physical and sexual health needs. They also highlighted the need for improved appointment booking arrangements for patients with SMI and the need to make mental health inpatient environments more conducive to good physical health. Greater prioritization of such health needs should be embedded in the culture and environment of mental health services. This will require clear strategic leadership and commitment from staff at all levels, backed by appropriate funding arrangements.

## Conclusion

The literature identified in this mapping review was restricted in volume, and few of the identified examples were described in great detail and fewer still were evaluated, raising questions about the replicability and generalisability of much of the existing evidence. A lack of evaluation and dissemination of local innovations makes it difficult for lessons learned locally to be shared across institutions and the wider health service.

Our approach was pragmatic and iterative in nature. Inevitably the process was less exhaustive and the outputs somewhat less detailed than might be expected from a full systematic review. Very few of the interventions described in the literature had any explicit theoretical basis, although aspects of this literature could be interpreted in light of existing theories of behaviour change. There might be an argument for undertaking a more interpretivist approach to exploring this literature. Such an investigation was outside the scope and resources of this mapping review.

Despite these limitations, some common themes did emerge from the evidence. Efforts to improve the physical health care of people with SMI should empower people (staff and service users) and help remove everyday barriers to delivering and accessing integrated care. In particular, the lack of confidence among many mental health practitioners about their own physical health care skills – and the need for training to address this – was raised by several respondents. Care co-ordinators/navigators may have an empowerment role by providing advocacy for service users in certain settings, and might themselves benefit from greater formal authority over care integration. All health professionals will need time to undergo training and to collaborate on patient care, which can be difficult in clinical settings with heavy caseloads. Management commitment to protect time and (where necessary) resources for such activities has been raised as a potentially worthwhile investment.

There is a need for improved communication between professionals and better information technology to support them, greater clarity about who is responsible and accountable for physical health care (such as cardiovascular monitoring), and awareness of the effects of mental health-related stigmatisation on the wider culture and environment in which services are delivered.

In 2013, the Mental Health Foundation concluded that good integrated care appears to be the exception rather than the norm, with isolated pockets of good practice, but overall dissatisfaction with progress being made overall. Our Advisory Group field experts gave the impression that this remained the state of affairs in 2015, describing a small number of high-profile programmes to address the physical health needs of people with SMI.

Ideally, future evaluations should be on a larger scale and use meaningful, validated and generalizable measures of success. In particular, evaluations need to be clear about which outcomes, facilitators, and barriers are likely to be context-specific and which might be generalisable. Wherever possible, service users should be involved in the design, conduct, and evaluation of programmes. For example, service users on our advisory panel identified scope for: improved appointment booking arrangements for patients with SMI; making mental health inpatient environments more conducive to good physical health; and greater attention to the sexual health of people with SMI. These concerns have received very little attention in the recent literature. There is scope for additional research on understanding why efforts to integrate physical health care needs for people with SMI succeed or fail, using qualitative or mixed-method techniques.
